# Vision System for Automatic On-Tree Kiwifruit Counting and Yield Estimation

**DOI:** 10.3390/s20154214

**Published:** 2020-07-29

**Authors:** Mohamed Lamine Mekhalfi, Carlo Nicolò, Ivan Ianniello, Federico Calamita, Rino Goller, Maurizio Barazzuol, Farid Melgani

**Affiliations:** 1 Metacortex S.r.l., Via dei Campi 27, 38050 Torcegno, Italy; carlo.nicolo@metacortex.it (C.N.); ivan.ianniello@metacortex.it (I.I.); federico.calamita@metacortex.it (F.C.); rino.goller@metacortex.it (R.G.); m.barazzuol@metacortex.it (M.B.); 2 Department of Information Engineering and Computer Science, University of Trento, Via Sommarive, 9, 38123 Trento, Italy; farid.melgani@unitn.it

**Keywords:** kiwifruit, fruit counting, yield estimation, computer vision fruit detection

## Abstract

Yield estimation is an essential preharvest practice among most large-scale farming companies, since it enables the predetermination of essential logistics to be allocated (i.e., transportation means, supplies, labor force, among others). An overestimation may thus incur further costs, whereas an underestimation entails potential crop waste. More interestingly, an accurate yield estimation enables stakeholders to better place themselves in the market. Yet, computer-aided precision farming is set to play a pivotal role in this respect. Kiwifruit represents a major produce in several countries (e.g., Italy, China, New and Zealand). However, up to date, the relevant literature remains short of a complete as well as automatic system for kiwifruit yield estimation. In this paper, we present a fully automatic and noninvasive computer vision system for kiwifruit yield estimation across a given orchard. It consists mainly of an optical sensor mounted on a minitractor that surveys the orchard of interest at a low pace. Afterwards, the acquired images are fed to a pipeline that incorporates image preprocessing, stitching, and fruit counting stages and outputs an estimated fruit count and yield estimation. Experimental results conducted on two large kiwifruit orchards confirm a high plausibility (i.e., errors of 6% and 15%) of the proposed system. The proposed yield estimation solution has been in commercial use for about 2 years. With respect to the traditional manual yield estimation carried out by kiwifruit companies, it was demonstrated to save a significant amount of time and cut down on estimation errors, especially when speaking of large-scale farming.

## 1. Introduction

Precision agriculture is projected to become the new normal among the farmers of the future [1,2,3,4,5,6,7,8]. A crucial concern in precision farming regards the postgrowth stage of the crops, more precisely yield estimation, which constitutes a pivotal step towards a proper set-up of the harvesting requirements (e.g., machinery, manpower, storage facilities, and transportation) while securing a well-posed marketing engagement (i.e., supply and demand).

The state of the art concerned with yield estimation and mapping survey several works, where the majority of which tends to rely on vision systems, owing to their noninvasive and crop-friendly nature. Moreover, imaging technologies are by far time and cost efficient with respect to manual counting and estimation. For instance, in [9], an image segmentation technique for fruit detection and yield mapping was put forth. Basically, it combines multilayer perceptrons and convolutional neural networks extended with contextual metadata for pixel-level fruit segmentation, which is accomplished with the well-known watershed technique. However, such pixel-wise segmentation schemes might be computationally prohibitive especially when addressing high-resolution images, notwithstanding the bottleneck of processing large-scale orchards. In [10], upright speeded-up robust features were leveraged for feature extraction in order to classify apple fruit based on mini-image tiles. Nevertheless, tile-based classification remains highly sensitive to the scale of the fruit. Moreover, if there are more than one fruit, they will be still counted as one fruit, which may cause a drastic underestimation. Conditional random field and sparse autoencoders were combined for image segmentation in [11], and fruit count was tackled (i) by converting the pixel count into fruit count or (ii) based on Hough transform to detect circular shapes. However, both strategies are limited in the sense that (i) pixel count does not change linearly with fruit count, whereas (ii) not all apple fruits exhibit a circular form (i.e., they might be viewed from the side or occluded by other fruits/leaves). Another apple yield estimation was presented in [12], where image segmentation based on empirical thresholds in the HSV color space was performed. Even though the results are plausible on the studied datasets, the robustness of the algorithm (i.e., the empirical thresholds) remains questionable when the sensor changes. In [13], color and texture features are combined and reduced by means of principal component analysis for pixel-wise segmentation, which as pointed out earlier, might come at high processing demands. An ensemble of machine learning classifiers was presented in [14,15,16,17]. However, when addressing large-scale orchards, large processing overheads are expected. The work presented in [18] addressed the problem of yield estimation in citrus orchards via image processing. The proposed solution consists in capturing an image of the citrus tree of interest by means of a DMC-ZS10 digital camera. Afterwards, the RGB and HSV color spaces of the shot image are leveraged to detect the color of the fruits (i.e., orange), followed by a watershed segmentation and a hole filling, which enables a final fruit counting. Although the proposed pipeline achieves interesting results, it remains limited in the sense that the yield estimation is carried out per tree, which is somewhat impractical since a typical orchard accommodates tens/hundreds of trees that need to be handled in an automatic fashion. In [19], a citrus yield estimation was presented. The system consists of a tractor, equipped with an optical camera, that drives along the orchard rows while taking images in a stationary mode. The acquired images are then converted into HSV space and further image-processing operations are applied. Although the described system is very practical, the performance of the fruit counting algorithm remains limited. Another orange fruit counting algorithm was proposed in [20]. The algorithm proceeds by a shadow removal via converting the acquired image into a LAB space. Next, the outcome is convolved with a variance mask and then converted into gray scale and thresholded in order to single out the overlapping fruits. Finally, a K-means segmentation is invoked for orange fruit detection. The system achieved an interesting overall detection score of 93.2%. Nevertheless, it still lacks the automatization property (i.e., estimating the yield for all the trees in a given orchard, rather than executing the task on a tree by tree basis). Apple fruit counting was addressed in [21], where a pixel-by-pixel classification is carried out via the Euclidean distance in the LAB space (only the L and B channels were considered). However, for the datasets adopted in the experiments, the disparity in color of the fruits with respect to the background is high, which suggests that a simple segmentation in the LAB color space (instead of a complicated and time-consuming pixel-wise classification) would potentially point out similar results. In [22], the well-known bag-of-words (BoW) model was adopted for pepper plants recognition and counting. The underlying idea of the presented scheme consists in combining the maximally stable color region features [23] and texture filters into a BoW signature with a support vector machine classifier. The achieved results are acceptable overall. However, instead of operating at pixel level as described earlier in [21], in [22], the classification is carried out inside a support window (measuring 40 × 90 pixels), which may help in cutting down the processing requirements when dealing with single images. However, it remains limited when the size of the target orchard is large. Deep learning, which has attracted remarkable interests in computer vision, in general, and object detection/recognition/tracking, in particular, was also adopted in [24] for tomato detection in images downloaded via Google image engine. In spite of the plausible accuracy which ranged within 80% and 100% for the considered images (100 samples), the applicability of such an algorithm in real time and in an automated way across a tomato orchard remains an open question. In [25,26,27], comprehensive surveys of existing works are presented. Yet, it can be drawn from the literature that the feasibility of a vision-based yield estimation system can be judged from two perspectives. The first component refers to the extent up to which the acquisition process is automated, whereas the second element has to do with the accuracy of the proposed yield estimation algorithm. Thus, both aspects are essential and go hand in hand for an efficient and automated yield estimation, which does not seem to be the case of the works conducted above. Another interesting fact is that several crops have received more attention in the literature (e.g., citrus crops), which might be subjective to technical or economic factors. In this respect, it is evident that the relevant state of the art is deemed rather poor when it comes to certain types of crops. For instance, kiwifruit is one of the crops that has undergone a steady increase in production worldwide in the last two decades and has reached unprecedented figures, with the three major exporters being China, Italy, and New Zealand. In particular, to the best of our knowledge, so far, there is no system that can carry out the kiwifruit yield estimation (i) for an entire orchard and (ii) in an automatic manner. The novelties of the present paper can be listed as follows:

- An automatic vision-based system for kiwifruit counting and yield estimation.
- To increase the robustness of the fruit detection pipeline, we emphasize on the kiwifruit tip instead of the whole fruit which may manifest various shapes, orientations, and occlusions.
- Instead of addressing only the fruit counting issue, we further assess the fruit yield and confront our findings with real data.
- Development of a user-friendly kiwifruit yield estimation interface that functions in two modes. The first one consists in a standalone application, whereas the second one is web based.

## 2. Materials and Methods

### 2.1. Prototype Description

**Figure 1:**
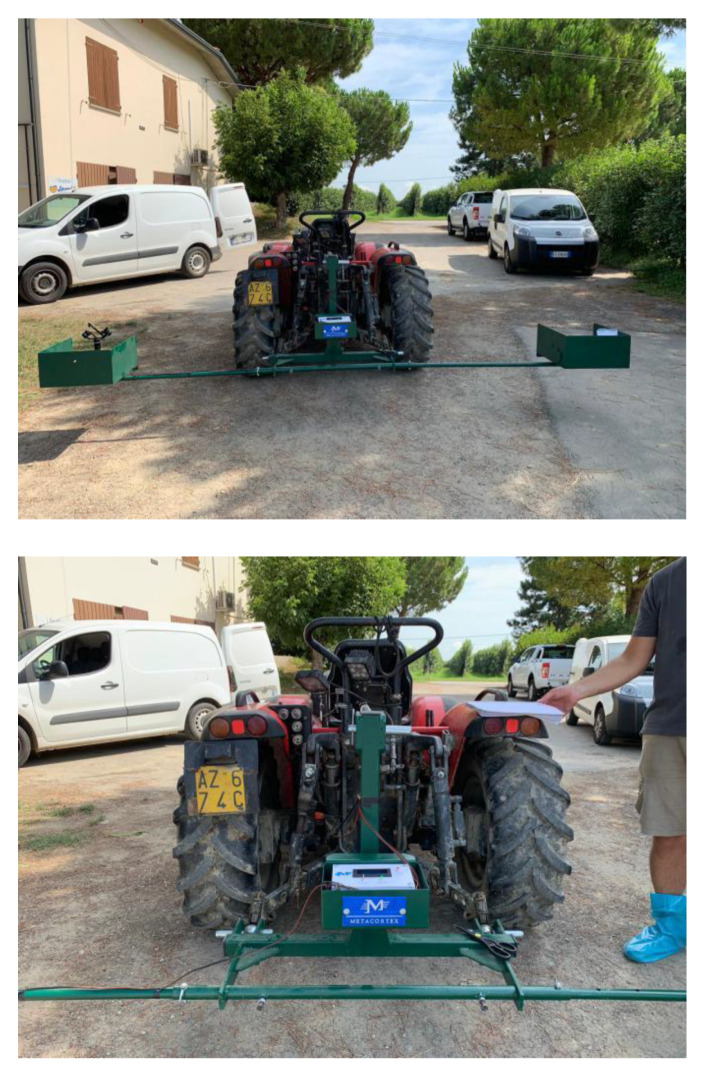
The horizontal bar carrying the other prototype components mounted at the rear of the minitractor.

**Figure 2:**
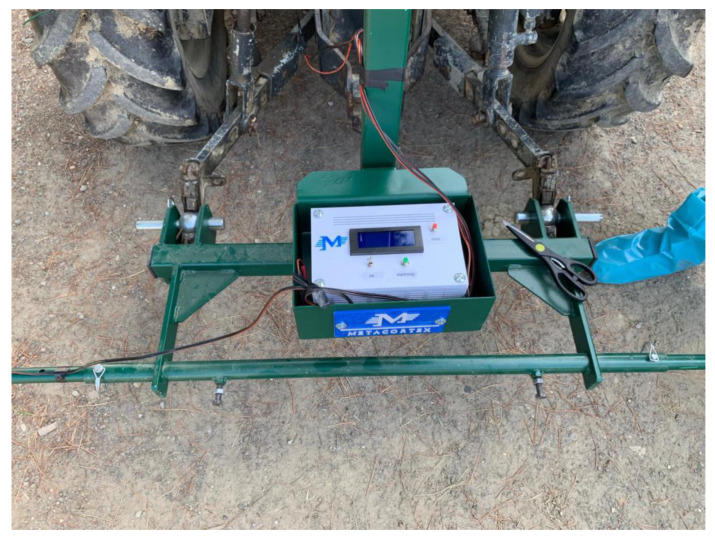
GPS module fixed on the bar.

**Figure 3:**
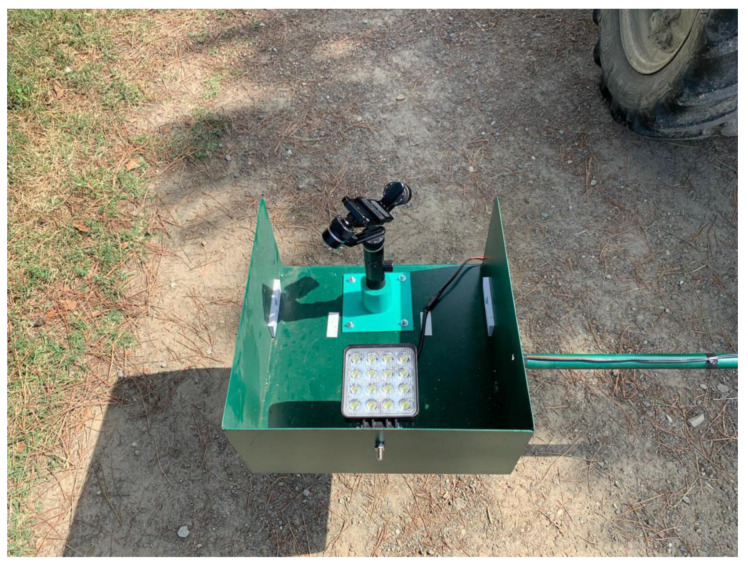
Gimbal, gimbal basis, and LED projector fixed on the bar and surrounded by a protective housing.

The prototype, as shown in Figure 1, is mountable on a minitractor and consists of several components:

- A horizontal bar that carries the other components, which can be attached/detached to/from the tractor. It is placed at the rear of the tractor by means of a three-point hitch.
- GPS module, as depicted in Figure 2, in order to save the location of the surveyed orchard, which is implemented with Arduino.
- LED projector (48 Watt, 3800 lm) powered with 12 V via the electric socket of the tractor. The LED is attached to the bar with a fastener and serves for illuminating the inner canopy of the kiwi trees due to the limited sunlight penetration through the leaves, as illustrated in Figure 3.
- Gimbal Feiyu G6 Plus to support and stabilize the camera against sudden tilts owing to the tractor’s vibrations as well as the rough nature of the terrain, as displayed in Figure 3.
- Support to attach the Gimbal on the bar (printed with a 3D printer “FlashForge Creator Pro” using a polylactic acid thread of 1.75 mm diameter), as shown in Figure 3 (i.e., the green piece).
- Camera (Sony Alpha 5100 of 24 MP) mounted on the support of the Gimbal.

**Figure 4:**
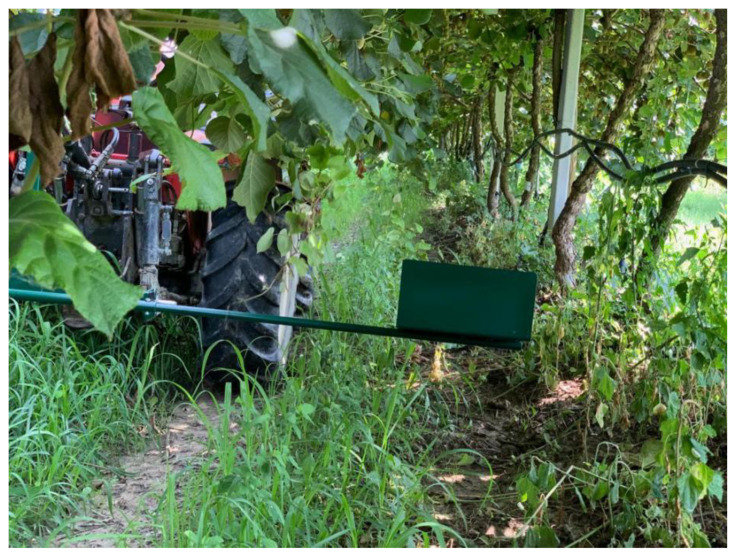
Camera position during the acquisition.

During the acquisition, the tractor is driven along the rows of the kiwi plants (Figure 4) and is maintained at a speed of roughly 2 km/h. The camera is mounted on the Gimbal (Figure 3) and directed upwards towards the tree canopy in order to capture the whole fruits at a frequency of 3 photos per second. The GPS module is utilized mainly to track the trail of the tractor and measures the current location every 5 s. It is worth noting that the camera can be placed either on the right end or on the left end of the bar. Various views of the prototype are provided below. Further, the end of the bar consists of a housing that protects the camera from being compromised by the surrounding leaves and twigs. 

### 2.2. Fruit Detection Method

#### 2.2.1. Detection Pipeline

**Figure 5:**
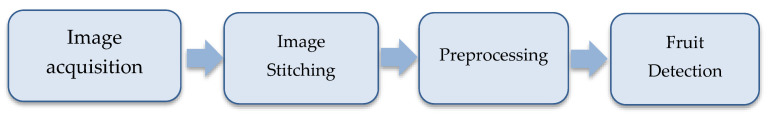
Proposed kiwifruit detection pipeline.

The proposed kiwifruit detection flowchart is given in Figure 5. After image acquisition along an orchard row, we proceed by image stitching in order to generate a unique nonrepetitive fruit image. Afterwards, this latter is preprocessed by suppressing the background and retaining the fruit regions. Finally, fruit detection and yield estimation are carried out on the preprocessed image. Details outlining each step are provided below.

#### 2.2.2. Image Stitching

Image stitching is carried out based on the Speeded-Up Robust Features (SURF) [28], which are interest keypoints that can be detected across a given image and describe local patterns that are robust to scale, illumination, and rotation changes. Thus, each keypoint is described by means of a 64-bin vector. On this point, given a pair of images acquired at two subsequent time instances, the stitching procedure is carried out over the following steps:

1. SURF feature extraction from both of the images.
2. SURF feature matching across both of the images based on Euclidean distance.
3. Apply the Random Sample Consensus (RANSAC) algorithm [29] on the matched feature set to estimate a homography matrix.
4. Apply an image warping transformation using the homography matrix that was estimated in the previous step.

**Figure 6:**
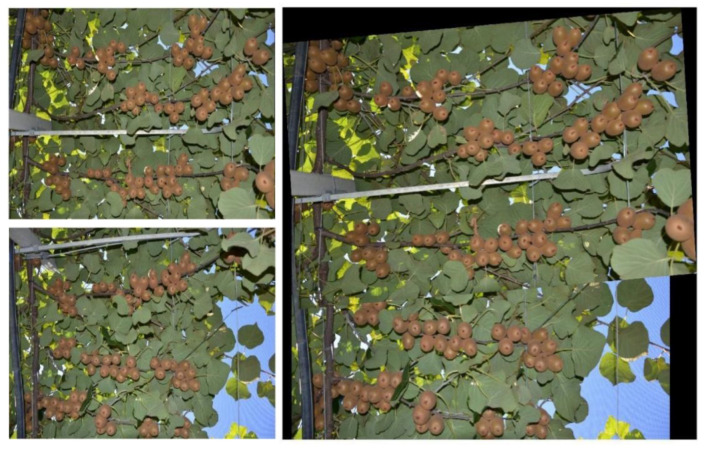
Image stitching example. (**Top left**): first image, (**Bottom left**): second image, (**Right**): stitched images.

An image stitching example is depicted in Figure 6.

#### 2.2.3. Preprocessing

**Figure 7:**
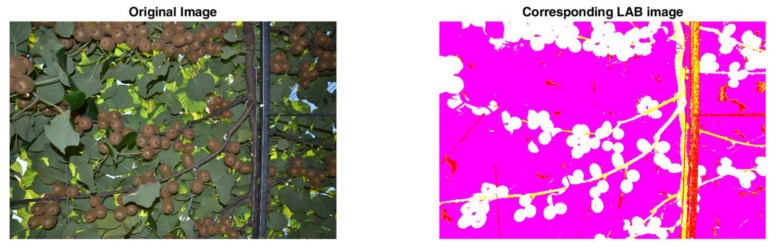
Example of an RGB image versus its respective LAB representation.

Prior to fruit detection, it is necessary to remove the background (i.e., consisting mostly of green leaves). This step is essential as to lessen the false positive detections as demonstrated later on in the experiments. Therefore, five stages are allocated as follows: first, the acquired RGB image is converted to the LAB space. This favors the suppression of a large amount of the background leaves as demonstrated in Figure 7. However, most of the foreground/background information is carried within the second channel, which represents the basis of the next operations.

Second, Otsu binarization [30] is applied on the “A” channel in order to generate a foreground–background mask. 

**Figure 8:**
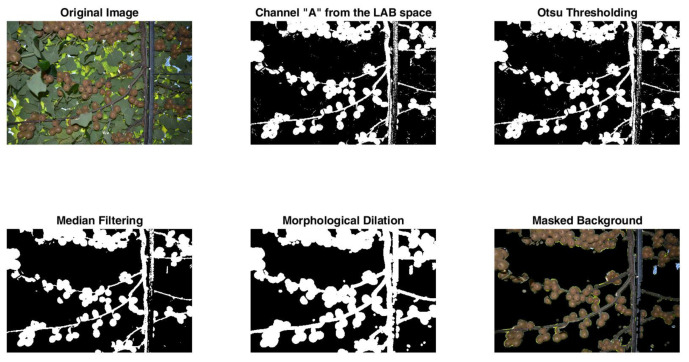
Image preprocessing example.

Third, the obtained mask is refined by a median filtering within a window of 25 × 25 pixels in order to alleviate the noise. Fourth, morphological dilation is applied to enlarge slightly the mask as to accommodate the fruits properly. Finally, the mask is applied on the original image in order to single out the fruits. An example depicting each step are displayed in Figure 8.

#### 2.2.4. Fruit Detection

**Figure 9:**
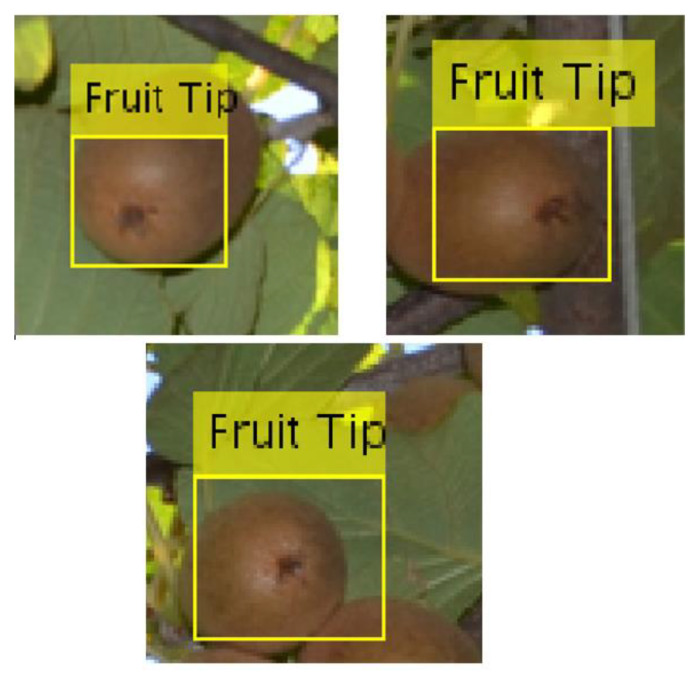
Examples of kiwifruit tip visibility.

It is evident that kiwifruits appear in a variety of sizes, orientations, and often occlude one another. These three bottlenecks render kiwifruit detection a rather difficult task. Therefore, in this paper, we propose another alternative, which capitalizes on the tip of the kiwifruit (i.e., the dark spot at the bottom of the fruit) rather than the whole fruit, owing to the fact that it is visible in most cases (provided that the camera is pointed upwards facing the bottom of the fruit canopy) as illustrated in Figure 9. 

**Figure 10:**
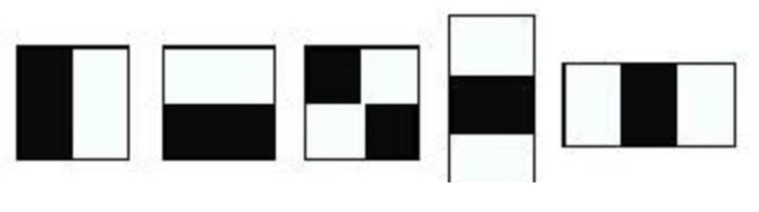
Several instances of rectangular Haar features. The feature response when applied on an image region equals to the sum of pixels under the shaded regions subtracted from the sum of pixels under the white region.

As far as the detection step is concerned, two prerequisites are envisioned, namely, (i) a plausible detection score and (ii) small processing overheads. Thus, one of the most suitable schemes in this regard is the well-known Viola and Jones object detection algorithm [31]. This technique was primarily introduced for face recognition. However, thanks to its robustness, simplicity, and high detection rates, it soon found its way to object detection, in general [32,33,34,35]. The underlying insight of Viola and Jones algorithm is to process the image at hand over a series of cascades, which consist of strong classifiers built upon a linear combination of weak classifiers. These latter are learned on a training set accommodating positive and negative samples. Interestingly, owing to the concept of detection over a cascade of classifiers, irrelevant objects within a given test image are mostly discarded at earlier stages, which enables the algorithm to emphasize on the image regions that are highly likely to contain the object of interest, which makes it an optimal technique for the kiwifruit detection endeavor (i.e., we would like the detection process to focus on kiwifruit tips without paying much attention to the irrelevant context/background). In what follows, we provide the essential steps underlining the Viola–Jones detection algorithm. The detection is performed by passing an ensemble of rectangular Haar features across the image. The role of those features is to capture potential image portions that correspond to the object of interest. Since such features are learnable depending on the form of the target objects, they can adopt a wide range of scales and shapes. Yet, learning such discriminative features may be highly prohibitive, especially when the objects exhibit some complications, notwithstanding the complexities characterizing the background regions. Therefore, in order to speed-up the process, there are three main steps featuring the Viola–Jones algorithm, namely, (i) integral image creation, (ii) training via AdaBoost, and (iii) cascading the classifiers. A Haar feature (Figure 10) may assume different sizes and forms and allows the calculation of image pixel disparities inside of it while sliding across the image. In order to lessen the processing burden, a so-called integral image is calculated (only once) prior to feature scanning and invoked afterwards each time the feature window changes size or form. 

For a given pixel in an image I, defined by the coordinates (x,y), its corresponding value within the integral image $I_{\mathrm{integral}}$ is the sum of the pixels within the top-left region of the pixel:

(1) $$I_{\mathrm{integral}}\left(x,y\right)=\underset{i,j}{\sum }I\left(i,j\right)\;i\leq x\;\&\;j\leq y\;$$

**Figure 11:**
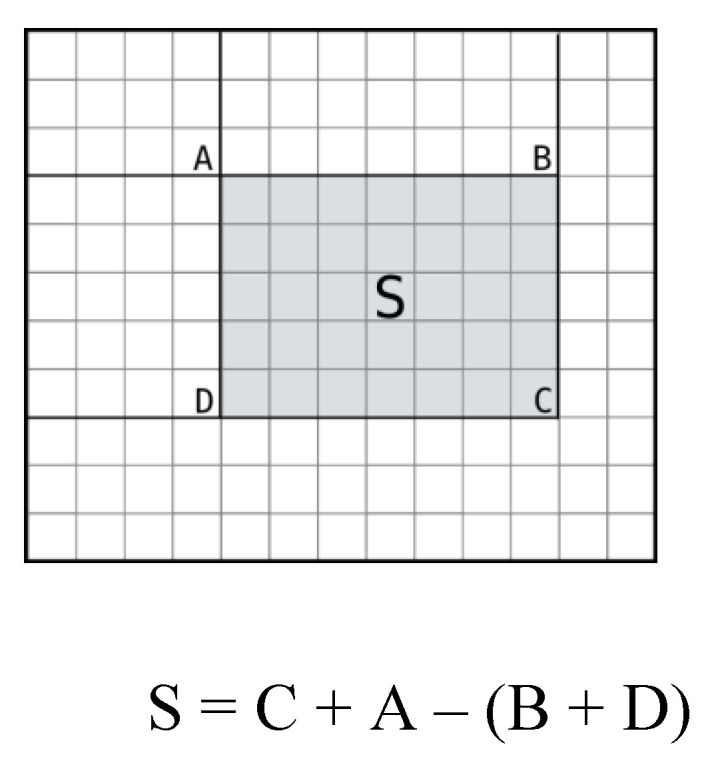
Example of fast pixel sum calculation from the integral image.

Once the integral image is calculated, the sum of pixels inside can be deduced by combining (i.e., summing and/or subtracting) the integral image values of neighboring pixels, an illustrative example is given in Figure 11.

Afterwards, a learning process, which selects rectangular features that best separate the positive and negative instances, is undertaken via a sliding detection window x across the image. Therefore, each rectangular feature is regarded as a weak classifier $h_{j}\left(x\right)$, which is defined by a feature $f_{j}$, a threshold $\theta_{j}$, and a parity $p_{j}$ to indicate the direction of the inequality sign:

(2) $$h_{j}\left(x\right)=\{\begin{array}{ll} 1 & if\;p_{j}f_{j}<p_{j}\theta_{j}\\ 0 & otherwise\ldots \ldots \end{array}$$

The boosting process, which consists in (i) the selection of a small subset of relevant rectangular features and (ii) the training of a strong classifier based on the ensemble of weak classifiers, is carried out by means of a variant of AdaBoost [36], as presented in Algorithm 1.
**Algorithm 1.** AdaBoost training procedure Given example images ($x_{i},y_{i})$, i=1,…,n, where $y_{i}=0,\;1$ for negative and positive examples, respectively.Initialize the weights $w_{1,i}=\frac{1}{2m},\frac{1}{2l}$ for $y_{i}=0,\;1$, for m negative and l positive samples, respectively.For t=1,…,T:Step 1. Weight normalization
(3)$$w_{t,i}=w_{t,i}/\overset{n}{\underset{j=1}{\sum }}w_{t,j}$$Step 2. For each feature j, a classifier $h_{j}$ is trained, whose error is calculated with respect to the weight, which enables the algorithm to emphasize on the most incorrectly classified instances in order to address them in further steps: (4)$$e_{j}=\underset{i}{\sum }w_{i}\left|h_{j}\left(x_{i}-y_{i}\right)\right|$$Step 3. Select the classifier that exhibits the smallest error.Step 4. Update the weights (5)$$w_{t+1,i}=w_{t,i}\beta_{t}^{1-c_{i}}$$where (6)$$c_{i}=\{\begin{array}{ll} 0 & \mathrm{correctly}\;\mathrm{classified}\;\mathrm{examples}\\ 1 & otherwise \end{array}$$and (7)$$\beta_{t}=\frac{e_{t}}{1-e_{t}}$$The final strong classifier is given by: (8)$$h_{j}\left(x\right)=\{\begin{array}{ll} 1 & \overset{T}{\underset{t=1}{\sum }}\propto_{t}h_{t}\left(x\right)\geq \;\frac{1}{2}\overset{T}{\underset{t=1}{\sum }}\propto_{t}\\ 0 & otherwise \end{array}$$

In order to increase the detection rate while keeping the processing demands in check, it is practical to frame the whole pipeline within a cascade framework in which an ensemble of subsequent classifiers is learned (using the above AdaBoost algorithm) so that positive windows detected by a certain classifier are forwarded to the next classifier, whereas negative windows are dropped immediately via a preset rejection rate, reducing thereby sharply the processing time. This strategy enables the algorithm to emphasize mostly on the potentially true positives while discarding potential false positives. For an in-depth analysis, the reader is referred to [31].

#### 2.2.5. Yield Estimation

**Figure 12:**
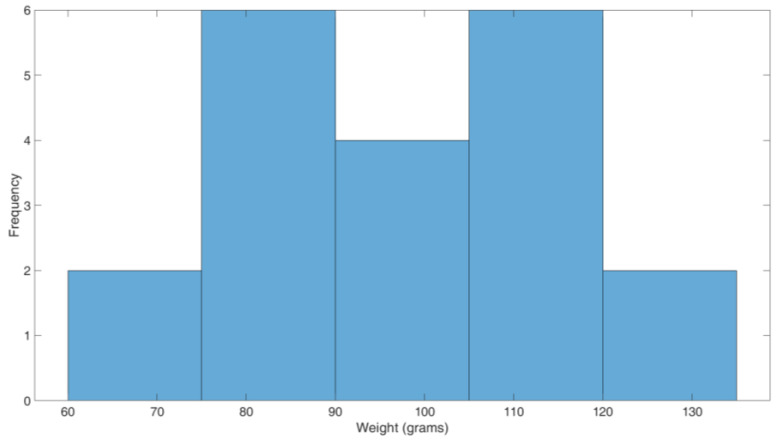
Kiwifruit weight distribution.

Yield estimation in terms of weight during the preharvest phase while the fruits are still on the tree (i.e., it may vary from late July till early October in Europe, depending on several factors such as rain and sun exposure, among others) can be a very complicated task. Thus, a straightforward approach is to multiply the total number of fruits by a representative weight. Therefore, we picked a bunch of 20 fruits (of difference sizes) from sparse locations across the fields under study and weighed them, whose distribution is shown in Figure 12. It can be observed that the fruit weight features a quasi-Gaussian trend on a five bins histogram. In our case, we consider the average, which amounts to 97.95 g as a representative weight. 

## 3. Experiments

### 3.1. Setup

**Figure 13:**
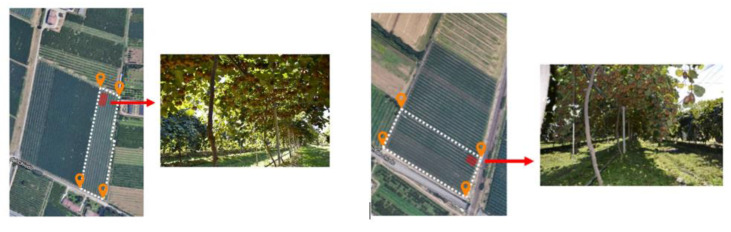
Experimental orchards depiction on Google Maps as well as on-site. (**Left**): orchard 1. (**Right**): orchard 2.

The experiments conducted in this work correspond to two kiwifruit orchards, which belong to Consorzio Frutteto, Cesena, Italy. The first orchard measures about 200 m × 50 m, whereas the second makes up roughly 150 m × 65 m, as shown in Figure 13. The kiwi vines are spaced by 2 m. The camera was placed at about 60 cm from the fruit canopy. First, we selected fifty images for the purpose of assessing quantitatively the fruit counting algorithm. Next, the full set of images across the two fields are used for a final yield estimation.

### 3.2. Dataset and Evaluation 

**Figure 14:**
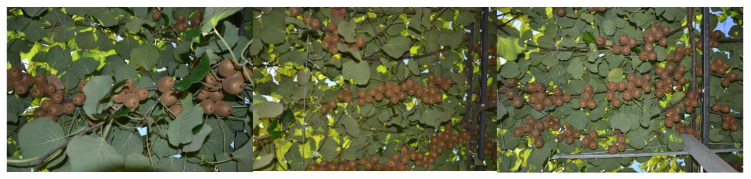
Image samples from the kiwifruit counting dataset.

In order to evaluate the performance of the proposed counting method, we selected 50 images. A few samples are depicted in Figure 14.

In the next three subsections, the dataset will be availed to study the impact of the preprocessing stage for different cascade numbers and the impact of the rejection rate. The average percentage error is used as an evaluation metric, which measures the absolute average counting error among the dataset images, given as

(9) $$APE(\%)=\frac{100}{N}\frac{\left|EC-AC\right|}{AC}$$

where *APE, N, EC,* and *AC* stand for the average percentage error, number of dataset images, estimated count, and actual count, respectively.

## 4. Results and Discussion

### 4.1. Effect of Preprocessing at Various Cascade Numbers 

**Figure 15:**
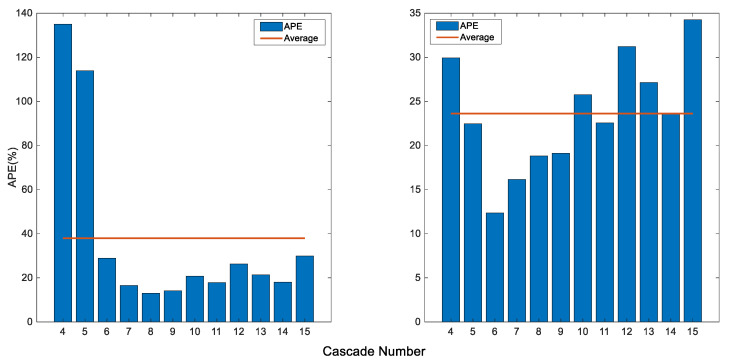
Effect of preprocessing with respect to cascade number. (**Left**): without preprocessing. (**Right**): with preprocessing.

We proceed by assessing the importance of the preprocessing phase in the proposed pipeline. In the meantime, we report the respective results for various cascade numbers; the scores are given in Figure 15.

**Figure 16:**
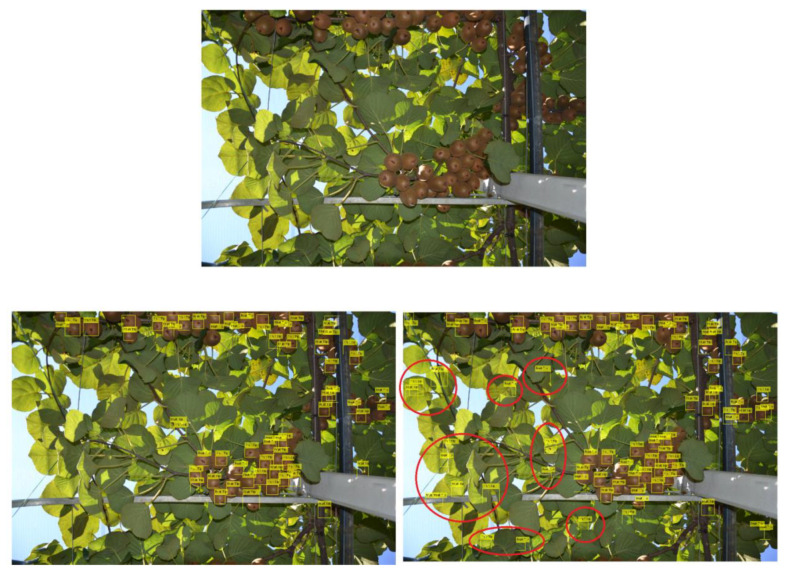
Example of kiwifruit detection with and without preprocessing using 6 cascades. (**Top**): original image. (**Bottom left**): detection with preprocessing. (**Bottom right**): without preprocessing where red circles indicate false alarms.

It can be seen from the figure that involving the preprocessing into the pipeline can incur improvements. For instance, with preprocessing, the best error rate of 12.35% is observed for 6 cascades, whereas the best score of 13.02% is noted at 8 cascades when preprocessing is discarded, which reveals that introducing preprocessing yields better results with fewer cascades (it is to remind that more cascades incite more processing demands). Moreover, on average over all the cascade numbers, a score of 23.62% is achieved when preprocessing is used, whereas discarding it scores an average of 38%, which further confirms the paramount role of preprocessing, especially when addressing a yield estimation of large orchard. We further illustrate this with a detection example in Figure 16, which confirms that excluding the preprocessing entails much more false positives. Thus, the next experiments will be carried out based on 6 cascades with preprocessing. 

### 4.2. Effect the Rejection Rate

The rejection rate is an essential parameter since it enables the control of the portion of false alarms (i.e., the false positives). It can be seen from Table 1 that a rejection rate of 20% stands out as the best heuristic. The results also show that going below or above that value, the APE rises, which can be explained by the fact that adopting higher values raises the chance detecting plenty of false alarms, whereas opting for smaller values comes at the risk of missing out on true positives (i.e., kiwifruits). A trade-off is thus necessary to balance the detection process. 

### 4.3. Yield Estimation

The proposed system was used to survey the yield of kiwifruits in both commercial orchards, and the results were confronted against the real data that was measured at the postharvest phase (all the fruits were weighed after the harvest) by Consorzio Frutteto, Cesena, Italy. On this point, our system overestimates the yield by 15% and 6% for both orchards, respectively. This, in fact, can be deemed an impressive yield estimation accuracy considering the challenging real-time acquisition scenarios as well as the fact that the detection pipeline consists of three major steps, namely, image stitching, preprocessing, and detection. Therefore, small errors in the earlier two steps may build up an error accumulation that has a direct impact on the fruit count, yet the yield estimation alike. It is to note that the proposed pipeline consumes about 1.6 s per image (of size 2000 × 3000 pixels) on a computer with an AMD Ryzen 3007 processor. This is an interesting feature as the detection algorithm does not require prohibitive processing facilities that may be necessary when implementing deep learning detection pipelines, for instance. The proposed system can also be extended to other crops provided that they manifest a characteristic that is unique and consistent among a large fruit set (i.e., the texture of the kiwifruit tip in our case) in order to guarantee small intraclass and large interclass variations. 

### 4.4. Graphical User Interface

It is essential to note that the whole system was developed with the intent to be operated by an end user (in our case, a technician with a solid background in farming). Therefore, it is necessary to keep the “how-to” simple and straightforward. Yet, we have built two modes in which the system can be utilized, namely, offline and online interfaces, which can be opted for upon the choice of the end-user.

**Figure 17:**
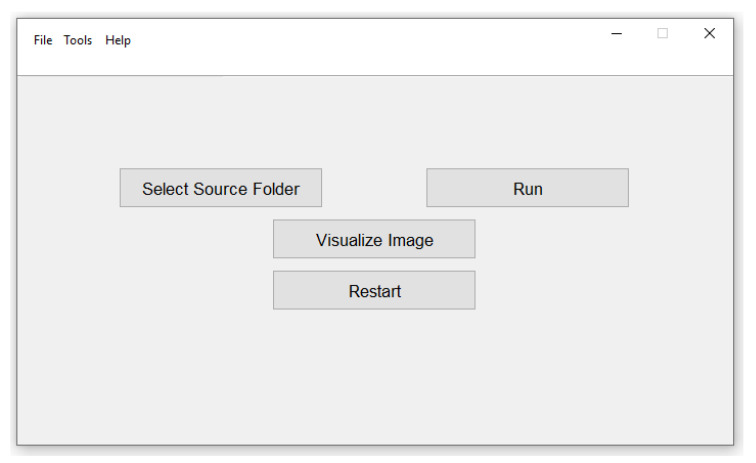
Offline kiwifruit yield estimation interface.

The offline interface (Figure 17) consists in a standalone executable application to be installed on any computer under any renown Windows or Linux distributions. The program has four buttons as follows:

- Select source folder: Serves for selecting the folder in which the acquired images are stored.
- Run: To launch the counting and yield estimation on the selected folder.
- Visualize image: To display fruit detection and counting instances.
- Restart: To launch another counting and yield estimation session.

It is to note that once the counting and yield estimation is launched, a progress bar shows up on the screen to indicate the status of the algorithm. Once the whole set of images is processed, the results will be summarized in a table at the bottom of the window, and a message is prompted asking the user whether or not to save the results in CSV format. 

**Figure 18:**
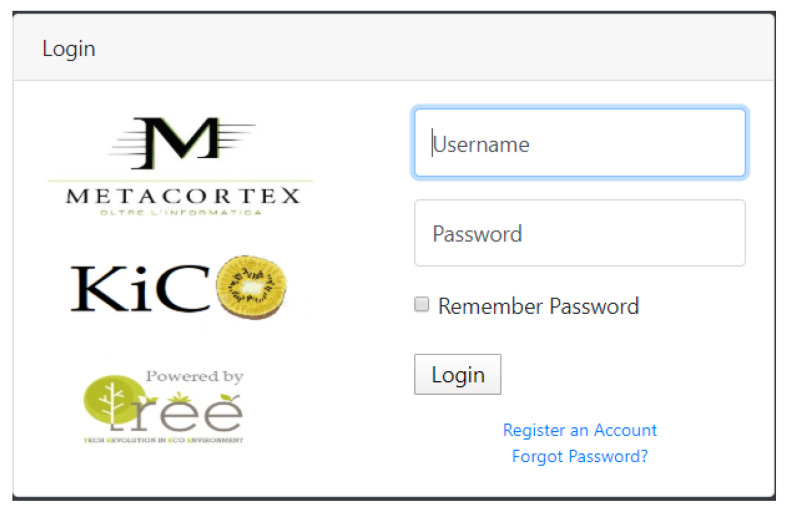
Login page of the web interface.

**Figure 19:**
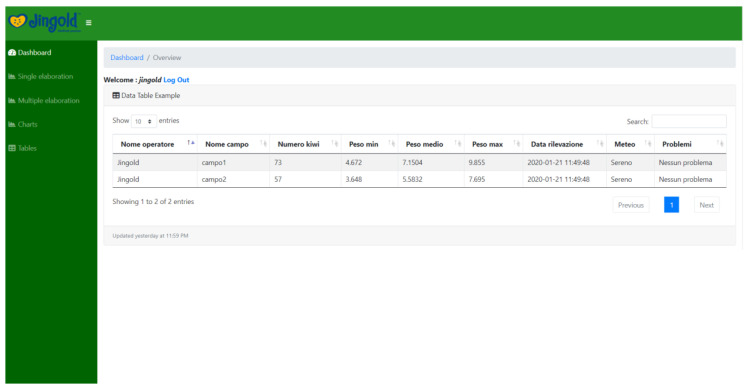
Web page-based kiwifruit counting and yield estimation.

**Figure 20:**
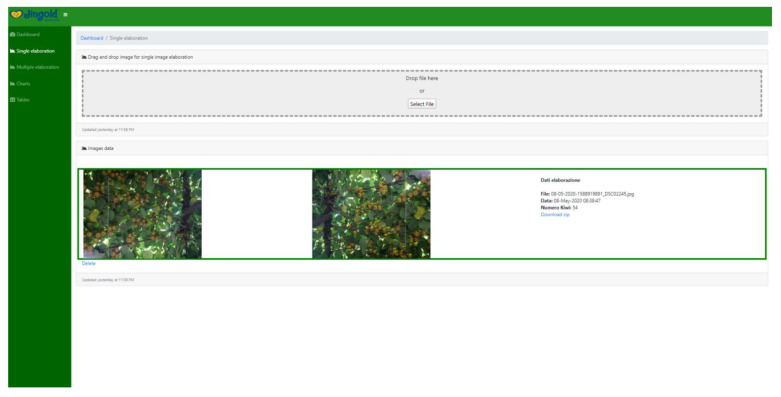
Web page-based kiwifruit counting instance display. (**Right**): input image. (**Left**): elaborated image and fruit count.

**Figure 21:**
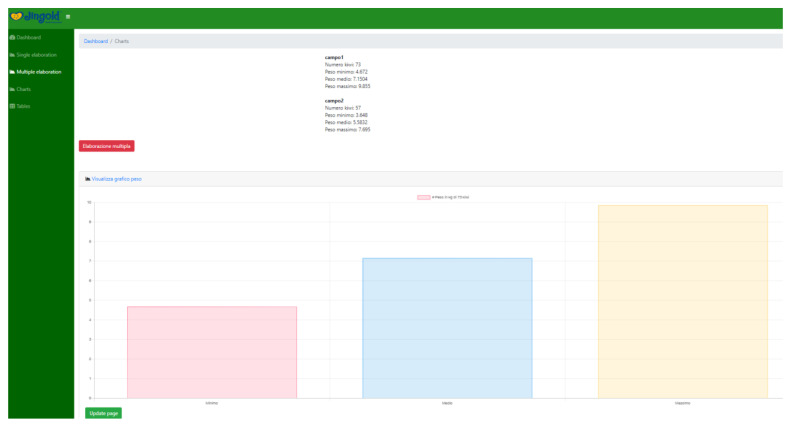
Yield estimation statistics.

The online interface (Figure 18, Figure 19, Figure 20, and Figure 21) offers the same functions. The user is required to input their credentials in order to log into their personal page where they can carry out fruit counting and yield estimation by uploading the images from a local folder (e.g., from an SD card). They can also produce CSV files and display various statistics. More interestingly, the statistics regarding the history of data accumulated over multiple estimations (e.g., over several years) can be displayed and interpreted.

## 5. Conclusions

We presented a computer vision system for automatic on-tree kiwifruit counting and yield estimation. Its main components consist of an optical sensor mounted on a minitractor via a horizontal bar. Further, a user-friendly interface was built in order to launch the counting and yield estimation tasks and save the results. Pros, Cons, and future improvements of the proposed system are provided below:

**Pros:**

- Fully automatic system that incorporates image acquisition, stitching, and counting in an end-to-end fashion.
- Robustness against fruit occlusion, size, and orientation changes.

**Cons:**

- The acquisition of images across a very large-scale orchard might take some time.
- Image acquisition is carried out on site, while fruit counting is performed off-site on a computer.

**Future improvements:** Although the proposed prototype is complete and accurate in its current form, we believe that potential hardware improvements can be introduced, mainly to minimize the on-site operational time. For instance.

- We are considering the adoption of another optical sensor in addition to the existing one in order to cut down the acquisition time by half.
- The yield estimation and fruit counting are an offline task in the current system. Thus, we plan to launch the whole process online on a minicomputer while the tractor drives across the orchard, which allows the human operator to access the results in real time.

## Figures and Tables

**Table 1:** Effect of the rejection rate.

Rejection Rate (%)	10	20	30	40	50
***APE* (%)**	20.16	12.36	24.23	38.12	41.48
